# Rapid Growth of Uropathogenic *Escherichia coli* during Human Urinary Tract Infection

**DOI:** 10.1128/mBio.00186-18

**Published:** 2018-03-06

**Authors:** Valerie S. Forsyth, Chelsie E. Armbruster, Sara N. Smith, Ali Pirani, A. Cody Springman, Matthew S. Walters, Greta R. Nielubowicz, Stephanie D. Himpsl, Evan S. Snitkin, Harry L. T. Mobley

**Affiliations:** a Department of Microbiology and Immunology, University of Michigan Medical School, Ann Arbor, Michigan, USA; b Department of Internal Medicine Division of Infectious Diseases, and Center for Microbial Systems, University of Michigan Medical School, Ann Arbor, Michigan, USA; UCLA School of Medicine

**Keywords:** ABU, ExPEC, PTR, UPEC, UTI, *in vivo* growth, plasmid segregation

## Abstract

Uropathogenic *Escherichia coli* (UPEC) strains cause most uncomplicated urinary tract infections (UTIs). These strains are a subgroup of extraintestinal pathogenic *E. coli* (ExPEC) strains that infect extraintestinal sites, including urinary tract, meninges, bloodstream, lungs, and surgical sites. Here, we hypothesize that UPEC isolates adapt to and grow more rapidly within the urinary tract than other *E. coli* isolates and survive in that niche. To date, there has not been a reliable method available to measure their growth rate *in vivo*. Here we used two methods: segregation of nonreplicating plasmid pGTR902, and peak-to-trough ratio (PTR), a sequencing-based method that enumerates bacterial chromosomal replication forks present during cell division. In the murine model of UTI, UPEC strain growth was robust *in vivo*, matching or exceeding *in vitro* growth rates and only slowing after reaching high CFU counts at 24 and 30 h postinoculation (hpi). In contrast, asymptomatic bacteriuria (ABU) strains tended to maintain high growth rates *in vivo* at 6, 24, and 30 hpi, and population densities did not increase, suggesting that host responses or elimination limited population growth. Fecal strains displayed moderate growth rates at 6 hpi but did not survive to later times. By PTR, *E. coli* in urine of human patients with UTIs displayed extraordinarily rapid growth during active infection, with a mean doubling time of 22.4 min. Thus, in addition to traditional virulence determinants, including adhesins, toxins, iron acquisition, and motility, very high growth rates *in vivo* and resistance to the innate immune response appear to be critical phenotypes of UPEC strains.

## IMPORTANCE

Uropathogenic *Escherichia coli* (UPEC) strains cause most urinary tract infections in otherwise healthy women. While we understand numerous virulence factors are utilized by *E. coli* to colonize and persist within the urinary tract, these properties are inconsequential unless bacteria can divide rapidly and survive the host immune response. To determine the contribution of growth rate to successful colonization and persistence, we employed two methods: one involving the segregation of a nonreplicating plasmid in bacteria as they divide and the peak-to-trough ratio, a sequencing-based method that enumerates chromosomal replication forks present during cell division. We found that UPEC strains divide extraordinarily rapidly during human UTIs. These techniques will be broadly applicable to measure *in vivo* growth rates of other bacterial pathogens during host colonization.

## INTRODUCTION

The urinary tract is the most common site of bacterial infection in humans (1). It has been estimated that at least 40 to 50% of women will experience a minimum of one symptomatic urinary tract infection (UTI) during their lifetime, with roughly 27 to 48% of affected women experiencing recurrent UTIs (2). In 2007, alone, UTIs resulted in 8.6 million physician visits, with women comprising 84% of the visits (3).

Lower UTI begins with bacterial colonization of the periurethral area by fecal contamination from the gastrointestinal tract, followed by ascension of bacteria through the urethra and into the bladder, causing cystitis. Unresolved cystitis may progress to an upper UTI, termed pyelonephritis, when bacteria ascend the ureters and enter one or both kidneys (4). Pyelonephritis, in some cases, progresses to bacteremia. Thus, during the course of an infection, a successful uropathogen must navigate dramatically different niches, including the gastrointestinal tract, periurethral area, urethra, bladder, ureter, kidney, and bloodstream.

*Escherichia coli* is the most common cause of UTIs (1, 5), with both symptomatic and asymptomatic infections most often being associated with specific uropathogenic *E. coli* (UPEC) sublineages. To elucidate the functional capacities that differentiate UPEC strains from *E. coli* strains that have distinct tropisms (e.g., gastrointestinal commensal lineages), UPEC strains were first studied *in vitro*, leading to the identification of distinguishing phenotypes, such as adherence and hemolytic activity, that contributed to the ability of the bacterium to infect and damage the host (6, 7). To more rigorously identify genes and pathways relevant to pathogenesis, our group, and others, began to employ animal models that mimic the human disease process (8). Increasingly sensitive methods have become available and are now being applied in these animal models or in human urine to understand the processes essential for uropathogenesis. These include competition experiments (9), application of signature-tagged mutagenesis (10, 11), identification of fitness genes by transposon sequencing (Tn-seq) (12–15), and measurement of the complete UPEC transcriptome during infection by microarray and transcriptome sequencing (RNA-seq) (16–18).

While these studies have provided a mechanistic understanding of what UPEC is doing during different stages of infection, it is far less clear how these functional dynamics relate to growth dynamics. In particular, while it has now become routine to quantify virtually every gene, protein, and metabolite produced by a bacterium, it has remained a challenge to probe the bacterial growth rate *in vivo*. In addition to providing necessary context for the proper interpretation of the aforementioned omics experiments, the measurement of the *in vivo* growth rate is essential (i) to understand the temporal dynamics of the infectious cycle (i.e., to determine how fast UPEC adapts to available nutrients in the host and how its capacity for rapid growth relates to host damage), (ii) to discern growth variation occurring in different compartments and anatomical sites (i.e., to determine whether growth is more rapid in the bladder or in urine itself), and (iii) to facilitate meaningful comparisons between different strains or species that may have different growth dynamics (i.e., to determine whether there are strain-dependent differences in growth rate).

In this study, we used UPEC, asymptomatic bacteriuria (ABU), and fecal isolates to test the hypothesis that UPEC isolates adapt more quickly to nutrient availability, grow more rapidly, and better survive the innate immune response within the urinary tract than other *E. coli* isolates. We employed two independent methods to assess the growth rate during infection: plasmid segregation and a sequencing-based method that enumerates bacterial chromosomal replication forks as a function of growth rate, known as the peak-to-trough ratio (PTR). We demonstrate that UPEC strains rapidly divide in both the murine and human urinary tracts and survive in the host.

## RESULTS

### Bladder colonization in the murine model is similar between *E. coli* isolates at 6 h, but fecal strains survive poorly 48 h postinoculation.

**FIG 1:**
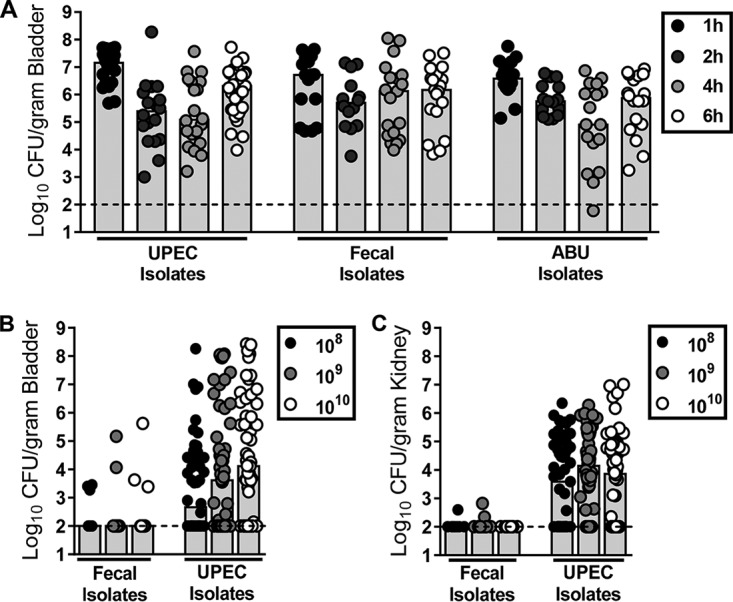
UPEC, fecal, and asymptomatic strains colonize the murine bladder at 6 hpi. (A) Bacterial recovery from bladders of mice transurethrally inoculated with 10^8^ CFU/ml UPEC (CFT073, UTI89, 536), fecal (EFC1, EFC2, EFC4, and EFC7), or ABU (PUTS37, PUTS58, PUTS59, and ABU83972) strains. Infections proceeded for 1 h (black circles), 2 h (dark gray circles), 4 h (light gray circles), or 6 h (open circles). (B and C) Bacterial recovery from the bladder (B) and kidneys (C) of mice transurethrally inoculated with 10^8^ (black circles), 10^9^ (gray circles), or 10^10^ (open circles) CFU/ml of fecal (EFC4 and EFC7) or UPEC (F3, F15, F11, F24, F54, CFT073, CFT269, CFT189, CFT204, and CFT325) isolates. Bladder and kidneys were harvested at 48 hpi. In panels A, B, and C, symbols represent individual mice and bars represent the median. *n =* 4 to 60. The limit of detection is 100 CFU/g. As an index of type 1 fimbrial expression, strain CFT073 agglutinated a suspension of yeast (*Sacchoromyces cerevisiae*) at a bacterial titer of 1:32. Strains ABU83972 and EFC7 failed to agglutinate yeast. Colonization and virulence gene data for each strain may be found in Fig. S1.

To test the hypothesis that UPEC isolates differentially establish and maintain infection in the urinary tract compared to fecal and ABU strains, we transurethrally inoculated C57BL/6 mice with 10^8^ CFU of 11 different *E. coli* strains representing UPEC (3 strains), ABU (4 strains), and fecal (4 strains) isolates. CFU per gram of bladder were determined 6 h postinoculation (hpi) (Fig. 1A). Every mouse was colonized, with median bladder values ranging from 7.6 × 10^5^ to 2.0 × 10^6^ CFU/g bladder. Thus, the fecal, UPEC, and ABU strains examined in this study can all survive in the urinary tract for at least 6 hpi.

10.1128/mBio.00186-18.2 FIG S1  Colonization and virulence gene data for each strain used in Fig. 1B and C. Bacteria were recovered from bladders (A) and kidneys (B) of mice transurethrally infected with 10^8^ CFU/ml fecal (EFC4 and EFC7) or UPEC (F3, F15, F11, F24, F54, CFT073, CFT269, CFT189, CFT204, and CFT325) isolates. Bladders and kidneys were harvested at 48 hpi. Symbols represent individual mice, and bars represent the median. *n =* 2 to 9. The limit of detection is 100 CFU/g. (C) The numbers of toxin and iron acquisition genes and fimbrial gene clusters present in each strain are listed, as confirmed by multiplex PCR. Download FIG S1, TIF file, 9.6 MB. Copyright © 2018 Forsyth et al. 2018 Forsyth et al. This content is distributed under the terms of the Creative Commons Attribution 4.0 International license.

Since it has been demonstrated that UPEC strains carry more virulence determinants than fecal strains (19, 20) (see Fig. S1 in the supplemental material) and cause persistent infections, whereas fecal strains do not, we transurethrally inoculated mice with either fecal or UPEC strains with inocula of 10^8^, 10^9^, or 10^10^ CFU. After 48 h, bladder bacterial burden was enumerated. All three inoculum concentrations resulted in bladder colonization by the UPEC isolates up to ~10^8^ CFU/g bladder; however, fecal strains survived poorly, with median values below the limit of detection of 100 CFU/g (Fig. 1B). Kidney colonization (Fig. 1C) was reflective of bladder colonization. These results indicated that fecal strains are unable to persist out to 48 hpi in the murine urinary tract following transurethral challenge, regardless of the inoculating dose. While resistance to the innate immune response, particularly neutrophil infiltration, may explain UPEC’s ability to colonize the urinary tract more successfully than fecal strains (21, 22), it is also possible that persistence may relate to the bacterial growth rate within urine and the host environment. For this reason, we sought to measure the bacterial growth rate within the urinary tract.

### *In vivo* doubling time for ABU strains is statistically longer than those for UPEC and fecal strains via plasmid partitioning.

**FIG 2:**
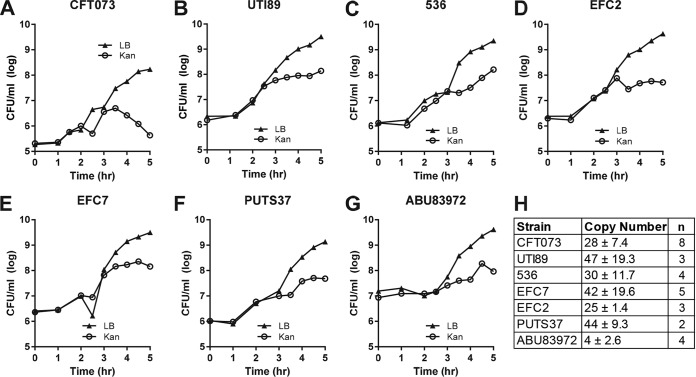
pGTR902 copy number is variable among EXPEC isolates. (A to G) Representative growth curves in LB diluted 1:100 (ABU83972) or 1:1,000 (CFT073, UTI89, 536, EFC2, EFC7, and PUTS37) from overnight cultures grown in LB supplemented with 1% l-arabinose and kanamycin (25 μg/ml). Cultures were grown at 37°C with aeration, and CFU per milliliter of pGTR902-containing bacteria and total bacteria (bacteria containing and those not containing pGTR902) were determined at 30-min or 1-h intervals by plating on LB agar containing 1% l-arabinose and kanamycin (25 μg/ml) (open symbols) and LB agar containing no antibiotic (closed symbols), respectively. (H) The copy number of pGTR902 in each isolate was calculated using the following equation: CFU/ml of pGTR902-containing bacteria at stationary phase/CFU/ml of pGTR902-containing bacteria in the inoculum. Values are mean ± standard deviation.

We utilized pGTR902, which replicates in the presence of l-arabinose (23), for estimation of bacterial growth rate in a collection of UPEC, fecal, and ABU isolates in the murine model of ascending UTI. To assess the growth rate, pGTR902 was introduced into UPEC isolate CFT073 by electroporation and cultured in LB medium with and without 1% l-arabinose. The total number of bacteria was enumerated by plating on LB agar, and the number harboring pGTR902 was determined by plating on LB agar containing kanamycin and l-arabinose. The presence of pGTR902 did not affect growth of CFT073 in LB broth with or without l-arabinose, and a decrease in the number of CFT073 cells containing pGTR902 was only observed in LB medium lacking l-arabinose (see Fig. S2 in the supplemental material). Comparisons between strains require precise determination of pGTR902 copy number in each strain. We therefore introduced pGTR902 into two additional UPEC isolates (UTI89 and 536), two fecal isolates (EFC7 and EFC2), and two ABU isolates (PUTS37 and ABU83972) and conducted *in vitro* growth experiments in LB medium without l-arabinose to calculate plasmid copy number based on plasmid segregation (Fig. 2). Plasmid segregation was observed in all seven strains (Fig. 2A to G), and average copy number was determined from 2 to 8 independent experiments per strain by dividing the number of pGTR902-containing bacteria at stationary phase by the number of pGTR902-containing bacteria present in the inoculum (Fig. 2H). Copy numbers differed between *E. coli* isolates, ranging from approximately 4 plasmids per bacterial cell in ABU83972 to approximately 47 plasmids per bacterial cell in UTI89. Plasmid segregation and copy number for CFT073, EFC7, and ABU83972 cultured in human urine were similar to that attained in LB (compare Fig. S3A to C in the supplemental material to Fig. 2A, E, G), suggesting that pGTR902 may segregate similarly within the urinary tract.

10.1128/mBio.00186-18.3 FIG S2  The presence of pGTR902 does not inhibit growth under permissive conditions. *E. coli* CFT073 harboring pGTR902, cultured in LB containing 1% l-arabinose and kanamycin (25μg/ml), was diluted 1:1,000 in LB with 1% l-arabinose and kanamycin (25 μg/ml) (open symbols) to allow pGTR902 replication or LB alone (closed symbols) to prohibit pGTR902 replication and incubated at 37°C for 5 h. The total CFU per milliliter and CFU per milliliter of pGTR902-containing bacteria were enumerated at 1-h intervals by plating on LB agar (circles) or LB containing 1% l-arabinose and kanamycin (25μg/ml) (squares), respectively. Representative growth curves are depicted. Download FIG S2, TIF file, 7.8 MB. Copyright © 2018 Forsyth et al. 2018 Forsyth et al. This content is distributed under the terms of the Creative Commons Attribution 4.0 International license.

10.1128/mBio.00186-18.4 FIG S3  pGTR902 copy number is stable during growth in urine. Shown are representative growth curves of (A) CFT073, (B) EFC7, or (C) ABU83972 containing pGTR902 grown in sterile human urine at 37°C with aeration. pGTR902-containing bacteria and total bacteria were determined at 30-min or 1-h intervals by plating on LB agar containing 1% l-arabinose and kanamycin (25 μg/ml) and LB agar containing no antibiotic, respectively. The copy number of pGTR902 was calculated with the formula CFU/ml of pGTR902-containing bacteria at stationary phase/CFU/ml of pGTR902-containing bacteria in the inoculum. The mean copy numbers from three replicates were 33 for CFT073, 32 for EFC7, and 11 for ABU83972. Download FIG S3, TIF file, 4.2 MB. Copyright © 2018 Forsyth et al. 2018 Forsyth et al. This content is distributed under the terms of the Creative Commons Attribution 4.0 International license.

To estimate growth rate *in vivo*, C57BL/6 mice were transurethrally inoculated with 10^8^ CFU/mouse of each *E. coli* isolate harboring pGTR902. Six hours postinfection was chosen as the ideal time point to harvest bladders for CFU as all seven isolates colonized to similar levels at this time (Table 1; see Fig. S4 in the supplemental material). Growth proportion, number of generations, and *in vivo* doubling time were estimated for each *E. coli* strain using total CFU recovered from the bladder of each mouse, the CFU of pGTR902-containing bacteria, and the experimentally determined plasmid copy number (Table 1). The *in vitro* doubling time of each strain during logarithmic growth in LB medium is shown for comparison. Overall, there were no statistically significant differences in bladder colonization, growth proportion, number of generations, or doubling time between each of the seven isolates. However, the average *in vivo* doubling time for the ABU group was statistically longer than the average for UPEC or fecal strains (*P* < 0.0001). Indeed, CFT073 and ABU83972 are the fastest- and slowest-growing strains, respectively, of all those tested using the plasmid segregation method. No significant differences in doubling time were observed *in vitro*. We conclude from this method that UPEC and fecal strains are capable of similarly high rates of growth within the mouse urinary tract, while ABU isolates exhibit longer doubling times *in vivo* than *in vitro*.

10.1128/mBio.00186-18.5 FIG S4  Colonization at 6 hpi is consistent among the majority of *E. coli* strains tested. Shown are the bacterial loads recovered from murine bladders 6 h after transurethral infection with 10^8^ CFU/ml of each of the strains used for calculation of growth rate via pGTR902. Symbols represent individual mice, and bars represent the median. *n =* 5 to 24. The limit of detection is 100 CFU/g. Means were compared using a two-way ANOVA with Tukey’s multiple-comparison test. No significant differences were found. Download FIG S4, TIF file, 10.6 MB. Copyright © 2018 Forsyth et al. 2018 Forsyth et al. This content is distributed under the terms of the Creative Commons Attribution 4.0 International license.

**TABLE 1:** Plasmid segregation as a method to estimate doubling time

Strain	Bladder log_10_ CFU^a^	Growth proportion^b^	No. of generations^c^	Doubling time (min)	
				*In vivo*^d^	*In vitro*^e^
UPEC isolates					
CFT073	6.6 ± 1.4	−3.6 ± 1.5	11.9 ± 5.0	36.3 ± 15.6	32.0 ± 4.8
UTI89	6.4 ± 0.5	−2.8 ± 0.6	9.3 ± 2.1	40.5 ± 8.7	26.5 ± 3.2
536	6.3 ± 0.7	−2.9 ± 0.5	9.6 ± 1.5	38.4 ± 6.3	28.0 ± 2.1
Avg	6.5 ± 1.1	−3.3 ± 1.3	11.0 ± 4.2	37.5 ± 13.2	29.4 ± 4.3
Fecal isolates					
EFC7	5.5 ± 0.6	−2.2 ± 0.6	7.1 ± 1.9	60.8 ± 3.3	27.5 ± 0.2
EFC2	6.0 ± 1.4	−2.4 ± 0.6	8.1 ± 2.0	46.5 ± 10.5	26.2 ± 0.4
Avg	5.8 ± 1.2	−2.3 ± 0.6	7.8 ± 1.9	53.7 ± 10.1	26.7 ± 0.8
ABU isolates					
PUTS37	5.8 ± 0.6	−2.1 ± 0.3	6.9 ± 1.1	53.0 ± 9.0	30.5 ± 1.1
ABU83972	4.2 ± 0.7	−1.3 ± 0.8	4.3 ± 2.7	112.6 ± 58.3	43.8 ± 17.9
Avg	5.0 ± 1.0	−1.7 ± 0.7	5.6 ± 2.4	82.8 ± 50.3^f^	39.4 ± 15.5

a C57BL/6 mice were transurethrally inoculated with 10^8^ CFU/mouse of each isolate harboring pGTR902. Bladders were harvested 6 h postinoculation for enumeration of total bacterial CFU and plasmid-containing CFU. Values are reported as the mean log_10_ CFU per gram of bladder tissue ± standard deviation for 4 to 21 mice per isolate.

b Growth proportion was calculated as follows: (log_10_ CFU/g of plasmid-containing bacteria/plasmid copy no.) − log_10_ total CFU/g.

c The number of generations for logarithmic bacterial growth *in vivo* was calculated as follows: growth proportion/−0.301.

d The doubling time for *in vivo* growth was calculated as follows: time postinoculation (min)/no. of generations.

e The doubling time *in vitro* during growth in LB was calculated as follows: time postinoculation (min)/[(3.3 × log_10_ CFU at inoculation)/log_10_ CFU at time postinoculation (min)].

f *P* < 0.0001 compared to UPEC and fecal isolates *in vivo* and ABU isolates *in vitro* by two-way analysis of variance (ANOVA) with Tukey test for multiple comparisons.

### Growth rate of CFT073 in human urine correlates with PTR.

**FIG 3:**
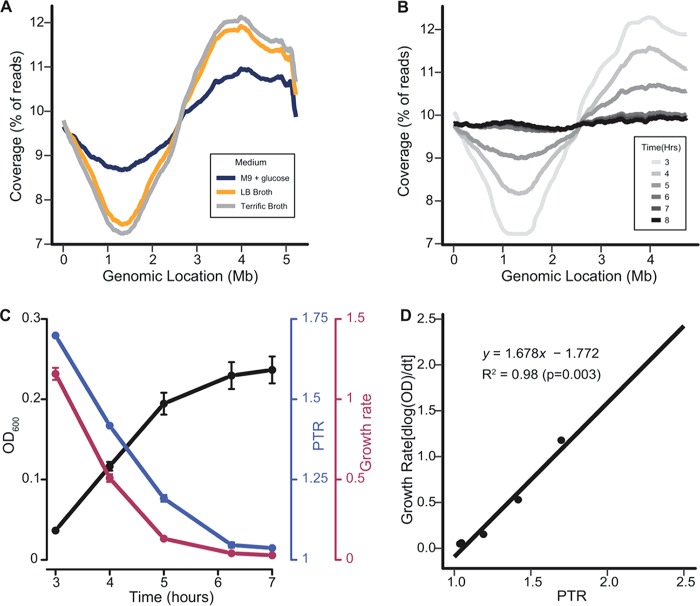
Growth rate is correlated with PTR *in vitro*. (A) *E. coli* CFT073 was inoculated into M9 supplemented with 0.4% glucose (blue), LB (yellow), and Terrific broth (gray) and grown to the mid-exponential phase (OD_600_ of 0.25, 0.55, and 0.67, respectively). DNA sequencing reads from each sample were aligned to the CFT073 chromosome (*x* axis), and the average coverage across the chromosome was calculated for replicate pairs (*y* axis). (B) DNA sequencing reads obtained from CFT073 grown in human urine were aligned to the CFT073 genome as in panel A. Average coverage from paired replicates is shown. (C) Triplicate OD_600_ measurements (black) at the corresponding time points from panel B. Growth rates (red) were calculated as the logarithm of the change in OD. PTR values (blue) from panel B are shown. Symbols represent the average, and bars represent the standard deviation. (D) A linear model was constructed using PTR and the growth rate of CFT073 in human urine.

As a second method to validate measurement of *in vivo* growth rate, we determined PTR. The assay is based on the principal that a rapidly growing bacterium will initiate multiple forks of replication about the origin of replication compared to the terminus of replication to keep pace with the rate of bacterial division. Thus, a faster-growing bacterium will have a higher PTR than a slower-growing bacterium (24). To test the technique, UPEC strain CFT073 was cultured with aeration in media that support different growth rates: M9 salts supplemented with 0.4% glucose, LB medium, and Terrific broth. Bacteria were collected during the mid-exponential phase of growth for each medium. Genomic DNA was isolated and subjected to Illumina sequencing. Sequence reads were aligned to the genomic sequence of CFT073 (Fig. 3A) (25). PTR was calculated using the following equation: % of reads at the origin/% of reads at the terminus. High PTR values were calculated for bacteria growing in rich media (LB medium and Terrific broth) (PTR = 1.62, 40.5-min doubling time, and PTR = 1.68, 40.0-min doubling time, respectively), and a low PTR value was calculated for bacteria growing in minimal medium (M9 salts supplemented with 0.4% glucose) (PTR = 1.27, 54.2-min doubling time) (Fig. 3A). As expected, bacteria growing rapidly in rich media display higher PTRs than bacteria growing slower in minimal medium.

Before determining the PTR of CFT073 *in vivo*, we first calculated the PTR of CFT073 cultured in pooled human urine. As expected, PTR values were high during the exponential phase and decreased in a stepwise fashion as the culture approached and entered the stationary phase (Fig. 3B and C). These data were used to construct a standard curve that correlated growth rate as measured by optical density at 600 nm (OD_600_) with PTR calculated at 3, 4, 5, 6, 7, and 8 h (Fig. 3D). A near perfect correlation of growth rate with PTR was observed (*R*^2^ = 0.98), allowing extrapolation of doubling time from a given PTR value.

### *E. coli* CFT073, ABU83972, and EFC7 have differential growth rates over time in the mouse model of UTI, as measured using PTR.

**FIG 4:**
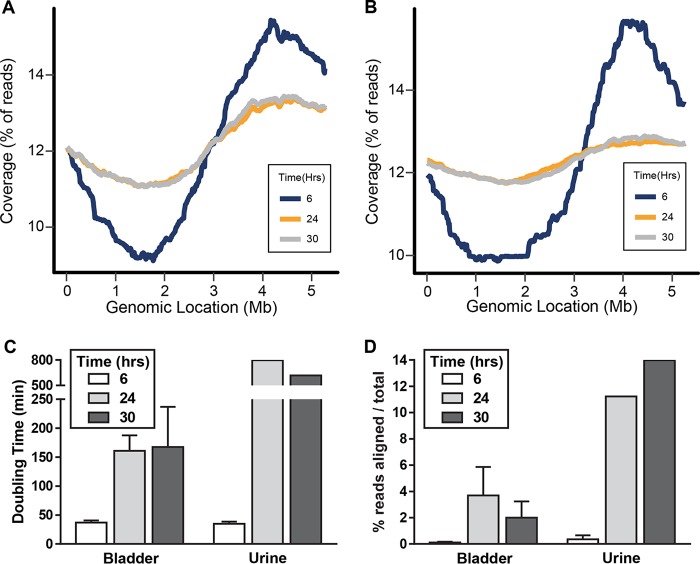
UPEC strain CFT073 growth is rapid at early time points during murine UTI. Mice were transurethrally inoculated with 10^8^ CFU/ml of *E. coli* CFT073. At 6 h (blue), 24 h (yellow), or 30 h (gray) postinoculation, bladder (A) and pooled urine (B) samples were processed to enrich for bacterial genomic DNA. DNA sequencing reads were aligned to the CFT073 genome (*x* axis), and the coverage across the chromosome was calculated (*y* axis). *n =* 3 to 4. (C) Doubling times for CFT073 in bladder and urine (*y* axis) were extrapolated using the formula from Fig. 2D. *n =* 3 to 4. Twenty-four-hour and 30-h urine samples from four mice were pooled for PTR determination. (D) The percentage of sequence reads from urine or bladder samples aligned to the CFT073 genome is shown (*y* axis) for the samples in panel C.

Mice were transurethrally inoculated with 10^8^ CFU *E. coli* CFT073. The urine and bladder samples of each mouse were collected at 6, 24, and 30 hpi. Bacteria were harvested from pooled urine (*n =* 4) by centrifugation, and genomic DNA was extracted. Bladder samples from individual mice were homogenized and enriched for bacterial cells using differential lysis (see Fig. S5 in the supplemental material), and DNA was isolated. DNA from urine and bladder preparations was subjected to Illumina sequencing. Enrichment of bladder homogenates for bacterial genomic DNA was critical to ensure that the threshold for accurate PTR determination, >0.2× genome coverage (see Fig. S6 in the supplemental material), was met. Treatment of bladder homogenates with mammalian cell lysis buffer had only a modest effect on bacterial viability compared to phosphate-buffered saline (PBS)-treated controls (see Fig. S7 in the supplemental material). PTR values determined for murine bladders (*n =* 3 to 4) predicted a high growth rate (PTR = 1.78, 36.9 + 3.8-min doubling time) at 6 hpi, slowing at 24 hpi (PTR = 1.21, 160 + 27-min doubling time) and 30 hpi (PTR = 1.22, 167 + 70-min doubling time) (Fig. 4A and C). Similarly, urine PTR values indicated a high growth rate (PTR = 1.75, 34.9-min doubling time) at 6 hpi, which slowed dramatically at 24 hpi (PTR = 1.09; 797-min doubling time) and 30 hpi (PTR = 1.10; 618-min doubling time) (Fig. 4B and C). The increasing fraction of sequence reads homologous to CFT073 at 24 and 30 hpi suggests that the bacteria reach a saturating population size within the nutrient-limited urinary tract, leading to decreased growth (Fig. 4D). This hypothesis is supported by an increase in bacterial load from ~10^6^ CFU/g bladder at 6 hpi to ~10^8^ CFU/g bladder at 24 hpi (see Fig. S8 in the supplemental material). An additional observation is the marked increase in the doubling time of the inoculum compared to the doubling time in the bladder at 6 hpi (119 min versus 36.9 min, respectively), indicating rapid adaptation of UPEC strain CFT073 to conditions within the murine host.

10.1128/mBio.00186-18.6 FIG S5  Differential lysis of bladder homogenates enriches for bacterial DNA. Quantification of bacterial and murine DNA present in homogenized bladders 6 h posttransurethral inoculation with 10^8^ CFU/ml CFT073. Bladder homogenates were treated to lyse mammalian cells prior to bacterial cell lysis or left untreated. Bacterial and murine DNA was quantified by quantitative PCR (qPCR) with primers homologous to *gapA* and the murine hemoglobin gene, respectively. Each symbol represents a single mouse. Bars represent median. *n =* 4. Download FIG S5, TIF file, 8.7 MB. Copyright © 2018 Forsyth et al. 2018 Forsyth et al. This content is distributed under the terms of the Creative Commons Attribution 4.0 International license.

10.1128/mBio.00186-18.7 FIG S6  Genome sequence coverage level required for accurate PTR. Shown are the PTR values calculated at the different genome sequence coverage levels given for a single replicate of CFT073 grown in pooled human urine *in vitro*, sampled over 6.5 h. The original coverage considering only the aligned sequence reads was down-sampled to different coverage values, and the pipeline was run on each of these down-sampled samples. PTR calculations showed very little change and remained consistent over a range of different coverages. The minimal coverage across the genome required for PTR to remain consistent was 0.2×. Download FIG S6, TIF file, 11.8 MB. Copyright © 2018 Forsyth et al. 2018 Forsyth et al. This content is distributed under the terms of the Creative Commons Attribution 4.0 International license.

10.1128/mBio.00186-18.8 FIG S7  Effect of mammalian cell lysis buffer on bacterial viability. Saturated cultures of *E. coli* strains (CFT073, EFC7, and ABU83972) incubated at 37°C with shaking were treated with mammalian cell lysis buffer (2 M Na_2_CO_3_, 1% Triton X-100 [pH 9.8]) or PBS, pelleted, resuspended, and plated for quantitative determination of CFU. Bars represent the mean; error bars represent standard deviation. *n =* 3. Means were compared using the Mann-Whitney test. *P* = 0.1. Download FIG S7, TIF file, 13.5 MB. Copyright © 2018 Forsyth et al. 2018 Forsyth et al. This content is distributed under the terms of the Creative Commons Attribution 4.0 International license.

10.1128/mBio.00186-18.9 FIG S8  Bacterial burden in murine bladder at 6, 24, and 30 hpi. Bacterial burden was enumerated from bladders of mice infected by being transurethrally inoculated with 10^8^ CFU/ml ABU83972, CFT073, or EFC7 and harvested at 6 (white), 24 (light gray), or 30 (dark gray) h postinoculation. Symbols represent individual mice, and bars represent the median. *n =* 3 to 4. The limit of detection is 20 CFU/g. Download FIG S8, TIF file, 7.9 MB. Copyright © 2018 Forsyth et al. 2018 Forsyth et al. This content is distributed under the terms of the Creative Commons Attribution 4.0 International license.

PTR values were also calculated for ABU strain ABU83972 and fecal strain EFC7 during murine UTI. However, because the ABU and fecal strain bacterial burden was not sufficient (<10^4^ CFU/g bladder [Fig. S8]) to obtain the genome coverage level required for the most accurate PTR determination, we can only define trends in growth rate. In contrast to CFT073, ABU83972 tended to maintain a relatively high growth rate in the bladder at 6, 24, and 30 hpi, with doubling times of 37, 31, and 47 min, respectively. Fecal strain EFC7 was found to have a moderately longer doubling time in the urine (72 min) at 6 hpi compared to ABU83972 and CFT073. Similarly, a modest increase in doubling time in the bladder was observed at 24 and 30 hpi (84 and 67 min, respectively) compared to 6 hpi (46 min). These results indicate that neither EFC7 nor ABU83972 reaches saturation in the bladder, yet ABU83972 maintains a high growth rate at 24 and 30 hpi, while EFC7 replicates more slowly at 24 and 30 hpi.

### PTR indicates rapid growth of UPEC isolates during human UTI.

By standardizing PTR measurement *in vitro* in human urine and *in vivo* using the mouse model, it was possible to estimate the growth rate of *E. coli* strains during uncomplicated UTI in women. Genomic DNA was extracted from urine that had been previously collected (and immediately stabilized with RNAprotect) from 38 female patients with significant *E. coli* bacteriuria (>10^5^ CFU/ml urine) and symptoms of cystitis and subjected to Illumina sequencing. Eight of these samples contained sufficient read coverage for analysis (see Fig. S9 in the supplemental material).

10.1128/mBio.00186-18.10 FIG S9  Quantification of DNA sequence reads across the genome of human isolates during uncomplicated UTI. PTR values were calculated from bacterial genomic DNA harvested from UTI patient urine. The raw sequence reads were mapped to the CFT073 genome (*x* axis), and PTR values were calculated from the sequencing coverage across the genome. The *y* axis is shown as a percentage of reads observed across the genome. Download FIG S9, TIF file, 11.8 MB. Copyright © 2018 Forsyth et al. 2018 Forsyth et al. This content is distributed under the terms of the Creative Commons Attribution 4.0 International license.

**FIG 5:**
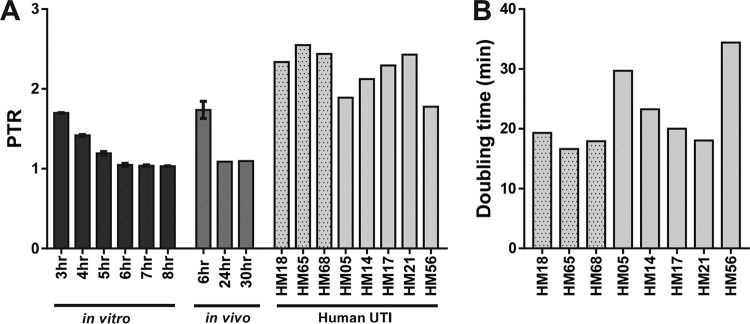
PTR of UPEC during human UTI is increased compared to *in vitro* growth or growth during murine UTI. (A) *E. coli* CFT073 containing pGTR902 grown *in vitro* in LB with 1% l-arabinose and kanamycin (25 μg/ml) was pelleted, washed in PBS, and diluted 1:1,000 into pooled human urine. Samples were taken hourly (3 to 8 h) and subjected to Illumina sequencing, and PTRs were calculated. *n =* 2. To calculate *in vivo* PTRs, mice were transurethrally inoculated with 10^8^ CFU/ml *E. coli* CFT073 containing pGTR902. The PTR of the inoculum of murine infection was 1.3, and the doubling time was 119 min. At 6, 24, or 30 hpi, bacterial genomic DNA from urine was sequenced. *n =* 4. PTRs of human urinary tract infections were calculated from bacterial genomic DNA harvested directly from infected human urine. (B) Doubling time (in minutes) extrapolated from the PTR of *E. coli* strains isolated during human UTI. In panels A and B, stippled bars represent individuals with no history of UTI. Each bar represents the mean. Error bars represent standard deviation.

PTR values ranged from 1.78 to 2.55, averaging 2.23 + 0.28 (Fig. 5A), which corresponds to very rapid doubling time estimates ranging from 16.6 to 34.4 min, averaging 22.4 + 6.4 min (Fig. 5B). While it did not escape our notice that these rates approached maximal growth rates for any *E. coli* strain under optimal *in vitro* culture conditions, the findings nevertheless indicate that *E. coli* strains are growing at surprisingly high rates during human infection of the urinary tract and faster than that for UPEC strain *E. coli* CFT073 during *in vitro* growth in human urine (36.0 ± 2.6 min) or within the mouse urinary tract (36.9 ± 3.8 min at 6 hpi). Isolates from women with a primary UTI (stippled bars) tended to have a shorter doubling time than isolates from women who suffered from recurrent UTI (plain bars) (Fig. 5B). However, this difference was not statistically significant (*P* = 0.19).

## DISCUSSION

Uropathogenic *E. coli* cells divide rapidly in both the murine and human urinary tracts, as measured by both a plasmid segregation method (mice only) and PTR (both mice and humans). At early time points postinoculation, ABU strains may be reliant on growth rate alone to successfully colonize as they often lack adhesins. Fecal strains, in general, do not carry a significant number of virulence determinants (19, 20) and did not colonize well at time points beyond 6 hpi. UPEC strains are relatively resistant to killing by the innate immune response at 48 h, as indicated by high bacterial loads recovered from the bladder over the entire time course of the infection studies. For the human studies, while we do not know precisely when *E. coli* entered the bladder, and thus the “hours postinoculation” are unknown, growth is uniformly rapid. These results suggest that UPEC strains are metabolically suited to grow rapidly in the urine within the bladder, even in the presence of a robust innate immune response. Indeed, damage to the host during infection, inflicted both by the bacterium and the host inflammatory response, likely releases additional nutrients to fuel this rapid growth.

We employed two techniques to measure growth rate *in vivo*. The first method followed plasmid segregation of a nonreplicating plasmid, pGTR902, developed for estimation of the growth rate of *Vibrio vulnificus* in skin lesions (23). One advantage of this technique is the ease of use in all *E. coli* strains tested. *E. coli* isolates transformed with plasmid pGTR902 were cultured *in vitro* in the presence of arabinose to drive replication of the plasmid and then washed and transurethrally inoculated into the murine urinary tract in which arabinose is absent. Rapid results were obtained within 24 h by plating on agar with and without l-arabinose and kanamycin to allow for differential enumeration of plasmid-containing bacteria. Limitations of the plasmid segregation method include the fact that it cannot be used in naturally occurring infections as the method requires experimental parameters to be defined. In addition, there is potential skewing of growth rate estimation if the plasmid does not properly segregate *in vivo* or if plasmid-containing bacteria are eliminated during infection.

The second method for determination of growth rate *in vivo* measured the peak-to-trough ratios for genomic DNA from *E. coli in vitro* or *in vivo*. This is a powerful tool for estimation of growth rate in an open system like the urinary tract and will not be skewed by loss of bacteria due to urination or ascension to the kidneys. It allows for estimation of bacterial growth rate in naturally occurring infections, not just experimental models. The limitations of the technique are that it is expensive and time-consuming compared to CFU determination. It requires sufficient read depth to accurately assess growth rate and requires assembly of reads on a genome scaffold (i.e., the strain’s genome must have been sequenced and assembled). Also, PTR estimates the average value for all bacteria in urine whether they are truly planktonic, adherent to exfoliated epithelial cells, or dead with genomic DNA undamaged.

Determinations for growth rates by the plasmid segregation method and PTR at 6 h postinoculation in the murine model agree well, with one notable exception. The doubling times for pyelonephritis strain *E. coli* CFT073 in the bladder were measured at 36.3 and 36.9 min by plasmid segregation and PTR, respectively. Indeed, the values were nearly identical. Values for UPEC type strains CFT073, UTI89, and 536 were also similar at 6 h in the bladder by plasmid segregation at 36.3, 40.5, and 38.4 min doubling times (average of 37.5 min), respectively. The latter strains (UTI89 and 536) were not tested by PTR. The growth rate of fecal strain *E. coli* EFC7 was slower than that of CFT073, with similar doubling times in the bladder of 60.8 and 46 min, as assessed by plasmid segregation and PTR, respectively. On the contrary, asymptomatic bacteriuria strain *E. coli* ABU83972 had estimated doubling times of 113 and 37 min by plasmid segregation and PTR, respectively. The reason for this dramatic difference is unclear but could be due to increased plasmid stability in the ABU strain versus those in the UPEC and fecal strains tested.

Using the plasmid segregation and PTR techniques, we were able to answer fundamental questions about UPEC biology in the urinary tract. For example, can we understand the temporal dynamics of the infectious cycle? That is, how fast does UPEC adapt to nutrient availability within its host? In a mouse inoculated with an overnight culture grown in LB, the doubling time was 119 min at the time of inoculation. Six hours after inoculation, the growth rate in the bladder increased to 36.9 min as it adapted to growth *in vivo*. This doubling time was consistent with estimates of 30 to 35 min in the first 8 h within intracellular bacterial communities (IBCs) within the bladders of mice experimentally infected with UPEC strain UTI89 (26). Surprisingly, despite the fact that fresh urine is constantly synthesized in the kidney and delivered to the bladder, thus refreshing the growth medium, *E. coli* appears to enter a stationary growth-like phase with slow doubling times by 24 hpi (161 min) and 30 hpi (167 min). During human infection, *E. coli* cells grow in the gastrointestinal tract and then contaminate the periurethral area and ascend the urethra to the bladder, and symptoms of cystitis are elicited between 24 h and 3 days postinoculation of the bladder (27, 28). It is at this point in the infection cycle that urine was collected from human patients, and PTR values were consistent with extraordinarily rapid growth (mean doubling time of 22.4 min). Thus, the human bladder appears to act more like a chemostat in which medium (urine) is refreshed constantly and outflow is accomplished by frequent urination. In the mouse bladder, urine may not be refreshed as rapidly as necessary to maintain exponential growth. This may reflect a fundamental difference between the murine model and human infection.

Differences in growth dynamics in the murine and human urinary tracts may also help explain differences in the expression of phase-variable type 1 fimbriae during infections in mice and humans. Selection for expression of these fimbriae is observed both under conditions of reduced oxygen and in the stationary phase of growth (29). Indeed, type 1 fimbriae are expressed during experimental murine UTI (30, 31), especially late in infection. This would be consistent with UPEC growth entering stationary phase at high CFU (~10^8^ CFU/g), likely due to limiting oxygen availability. On the other hand, several studies have found the orientation of the *fim* promoter is more often in the “off” position when examined directly from the urine of infected women (17, 18, 32). That *E. coli* cells examined in the urine collected and stabilized immediately have growth rates consistent with exponential growth may explain why a substantial percentage of the isolates are not expressing type 1 fimbrial genes during the human infections. Higher oxygen tension in the human bladder compared to the mouse bladder may also help to explain this, but has not been measured.

We can also ask whether there is growth variation occurring in different anatomical sites. When PTR values during murine infection were compared between the urine and the bladder for UPEC strain CFT073, similar doubling times were observed at 6 hpi (34.9 and 36.9 min, respectively); however, at 24 and 30 hpi, doubling times were dramatically longer in the urine than in the bladder. Given that PTR measures the average of all bacteria whether planktonic, adherent, or intracellular, bacteria in the bladder are replicating faster beyond 6 hpi. It is possible that concentrations of nutrients are higher in the bladder at later time points due to damage to the epithelium by the action of bacterial cytotoxicity and the process of inflammation.

Further, we may ask if meaningful comparisons can be made between strains with potentially different growth dynamics. We know well that UPEC strains display tremendous heterogeneity with respect to genes present in pathogenicity islands beyond the conserved base genome found in commensal strains (19, 20, 33). Indeed, this heterogeneity is displayed by the strains from women with symptoms of cystitis (17). Sequencing of these strains (34) revealed differences in genome size and the presence of a wide variety of accessory genes necessary to colonize the urinary tract (i.e., different combinations of adhesins, iron acquisition systems, and toxins). Consequently, the human UPEC strains displayed variation in growth in human urinary tract infection (Fig. 5). Indeed, expression of type 1 fimbriae may provide an alternative explanation for the differences observed in bladder colonization between strain types. Fecal strain EFC7 and asymptomatic strain ABU83972 do not agglutinate yeast (data not shown), indicative of a lack of expression of functional type 1 fimbriae.

*E. coli* transcriptome profiles from the urine of patients with urinary tract infection, described in three reports using either microarray technology (18) or RNA sequencing (17, 35), are consistent with the ability to achieve rapid bacterial growth *in vivo* compared to *in vitro* culture (Fig. 5). In one study from urology clinic patients with *E. coli* bacteriuria (18), the most highly expressed bacterial genes in urine were those encoding ribosomal protein subunits. Ribosomal genes represented between 24 and 54% of the top 50 upregulated genes for the eight *E. coli* isolates compared to gene expression by the same isolates in LB cultures. Selected nonribosomal genes, upregulated during UTI, were also consistent with rapid growth *in vivo*, including those required for translation, the F_o_F_1_ ATPase, fatty acid biosynthesis, and protein folding and secretion. *E. coli* from the urine of elderly patients with UTI (35) upregulated 202 genes compared to *in vitro* culture in rich medium. Twenty percent of these upregulated genes were involved in translation (ribosomal protein genes) or ATP synthesis, all indicative of rapid growth. Finally, in a transcriptome study of *E. coli* in the urine of patients with uncomplicated cystitis (17), similar genes, including those encoding ribosomal proteins, were highly upregulated during infection compared to *in vivo* growth in human urine or LB medium.

We have shown that the mean PTR value from *E. coli* strains collected during active uncomplicated UTIs is 2.0 ± 0.5 (Fig. 5A). Although this value reflects a very high growth rate and exceeds the range of PTR values obtained *in vitro* used to establish a standard curve (Fig. 3), it does not exceed the range of PTR values found in previous studies. Korem et al. reported a PTR of 2.6 for commensal *E. coli* (K-12 NCM 3722) during the exponential phase *in vitro* and PTR values ranging from 1 to 2.6 for *E. coli* in human fecal samples (36). One study comparing the growth rate of *E. coli* strains on infant skin, mouth, and gut samples found PTR values ranging from 0.9 to 2.2, with PTR values in the skin and mouth significantly higher than in the gut (37). Brown et al. found *E. coli* in the gut of a premature infant having a PTR of 1.91. In the same study, PTR values for all species present ranged from 1.2 to 2.6 in the premature infant microbiome and 1.1 to 2.1 in the adult microbiome (38). Thus, our reported PTR values for UPEC in human UTI are within the bounds of published microbiome studies.

Certainly, future questions remain. For example, do UPEC strains persist by employing immune evasion or altering metabolic strategy at later time-points? What prevents UPEC strains in humans from progressing to a systemic infection with higher frequency than is observed clinically given the high growth rate calculated here? In all, however, this study highlights the use of PTR to estimate relative rates of growth in murine and human urinary tract infections and reveals that uropathogenic *E. coli* strains are replicating at a surprisingly high rate during human infection. This technique should be widely applicable for measurement of bacterial growth rates during infection.

## MATERIALS AND METHODS

Extended materials and methods can be found in Text S1 in the supplemental material.

10.1128/mBio.00186-18.1 TEXT S1  Supplemental methods. Download TEXT S1, DOCX file, 0.1 MB. Copyright © 2018 Forsyth et al. 2018 Forsyth et al. This content is distributed under the terms of the Creative Commons Attribution 4.0 International license.

### Ethics statement.

Urine collection was approved by the University of Michigan Institutional Review Board (HUM00004949). Informed consent for collection of urine specimens from women attending the University Health Services was approved by the Michigan Institutional Review Board (HUM00029910) (17). All animal protocols were approved by the Institutional Animal Care and Use Committee (IACUC) at the University of Michigan Medical School (PRO00005052).

### Bacterial strains, plasmids, and culture conditions.

*E. coli* UPEC strains CFT073 (39), UTI89 (40), and 536 (41), commensal fecal strains (EFC1, EFC2, EFC4, and EFC7 [39]), and asymptomatic bacteriuria strains (PUTS37, PUTS58, PUTS59 [42], and ABU83972 [43]) were used in this study. Genomic DNA of eight UPEC strains (HM05, HM14, HM1, HM18, HM21, HM56, HM65, and HM68) was isolated from the urine of women with cystitis and sequenced. Strains were cultured in LB broth, Terrific broth, M9 minimal medium, or LB agar.

### Segregation of plasmid pGTR902 *in vitro.*

Plasmid pGTR902, transformed into *E. coli* strains, was used to estimate growth rate as previously described (23).

### Murine model of ascending UTI.

Six- to eight-week-old female C57BL/6 mice (Envigo) were infected as previously described (44, 45).

### Segregation of plasmid pGTR902 *in vivo.*

Bacteria transformed with plasmid pGTR902 were cultured overnight with 1% arabinose and kanamycin (25 μg/ml), harvested, and washed. A total of 10^8^ CFU were transurethrally inoculated into the mouse bladder. At 6 hpi, bladders and kidneys were removed, homogenized, and plated onto LB agar with and without 1% l-arabinose and kanamycin (25 μg/ml). The calculations used to determine the *in vivo* number of generations have been described previously (23). We have made the logical extension of these calculations to determine the doubling time.

### Estimation of *in vitro* and *in vivo* growth rates via PTRs.

Genomic DNA, isolated from bacteria harvested from culture medium, bladders, and urine of infected mice and bacteria in the urine of women with *E. coli* bacteriuria, was subjected to Illumina sequencing. PTRs were calculated using the peak and trough location with maximum and minimum values from the resulting smoothed coverage (36, 46–48).
