# Supplementary figures and images for: Rapid Growth of Uropathogenic Escherichia coli during Human Urinary Tract Infection
Source: mBio. 2018 Mar 6;9(2):e00186-18. doi: 10.1128/mBio.00186-18 (PMC5844997; doi:10.1128/mBio.00186-18)

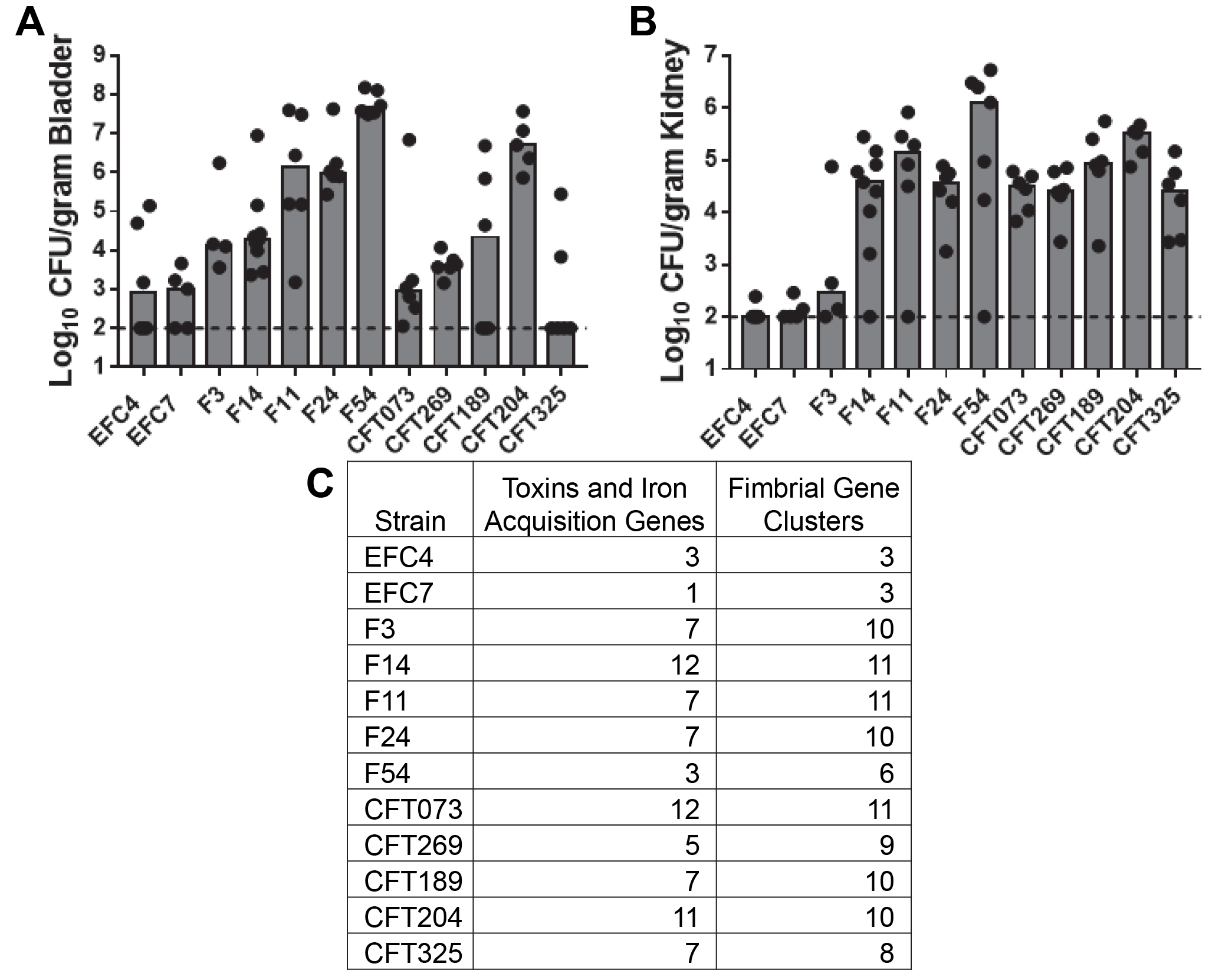

Supplement: FIG S1 [file mbo001183754sf1.tif]

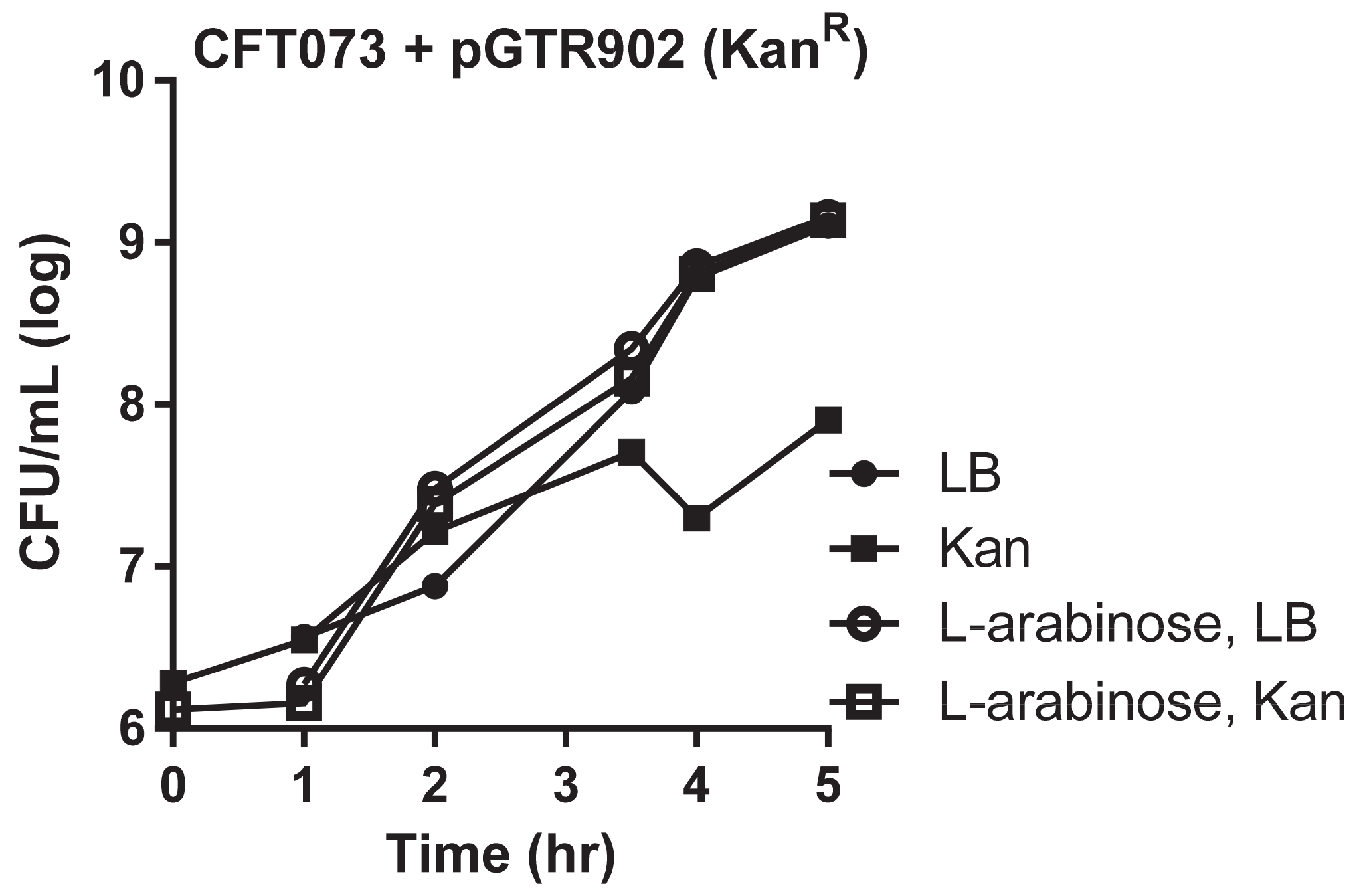

Supplement: FIG S2 [file mbo001183754sf2.tif]

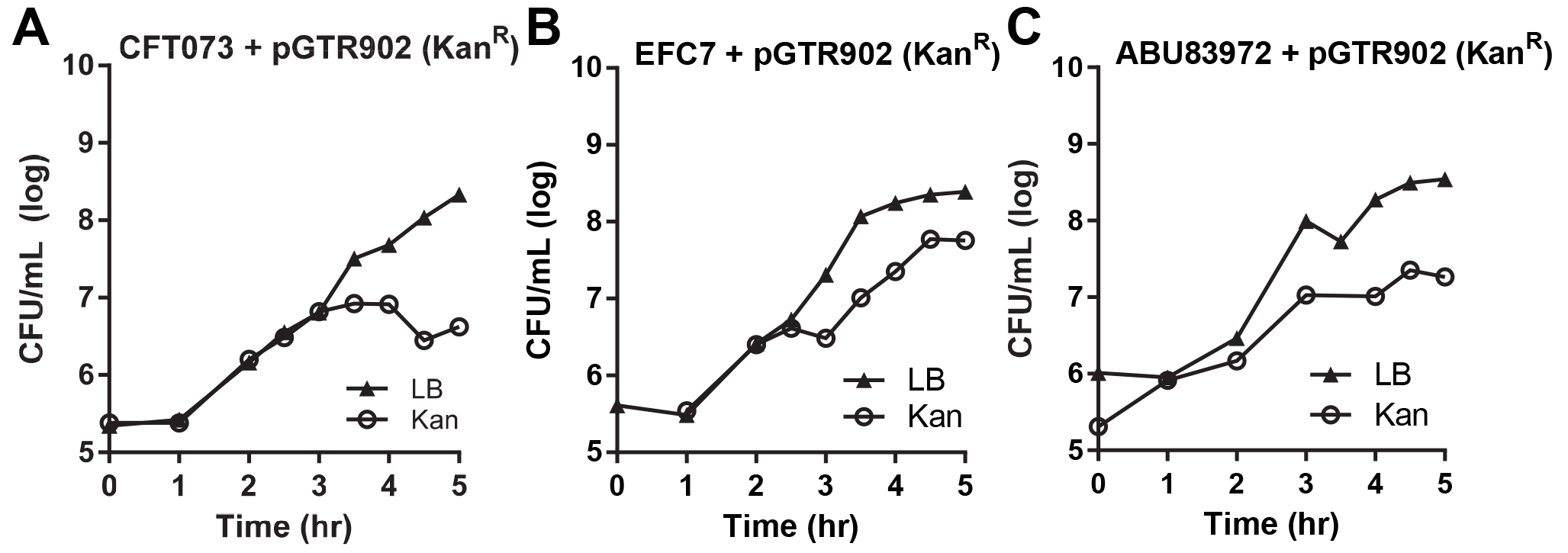

Supplement: FIG S3 [file mbo001183754sf3.tif]

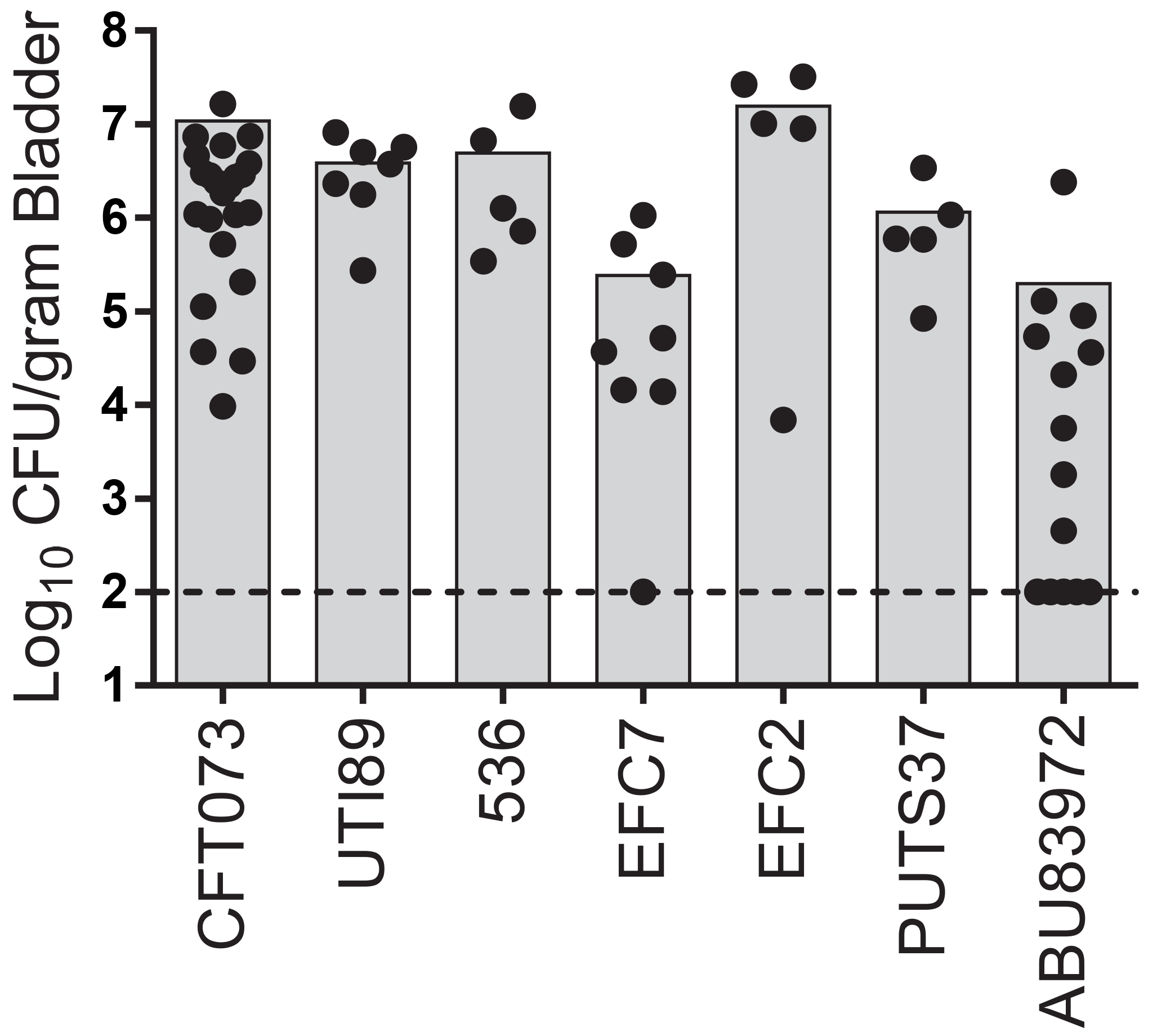

Supplement: FIG S4 [file mbo001183754sf4.tif]

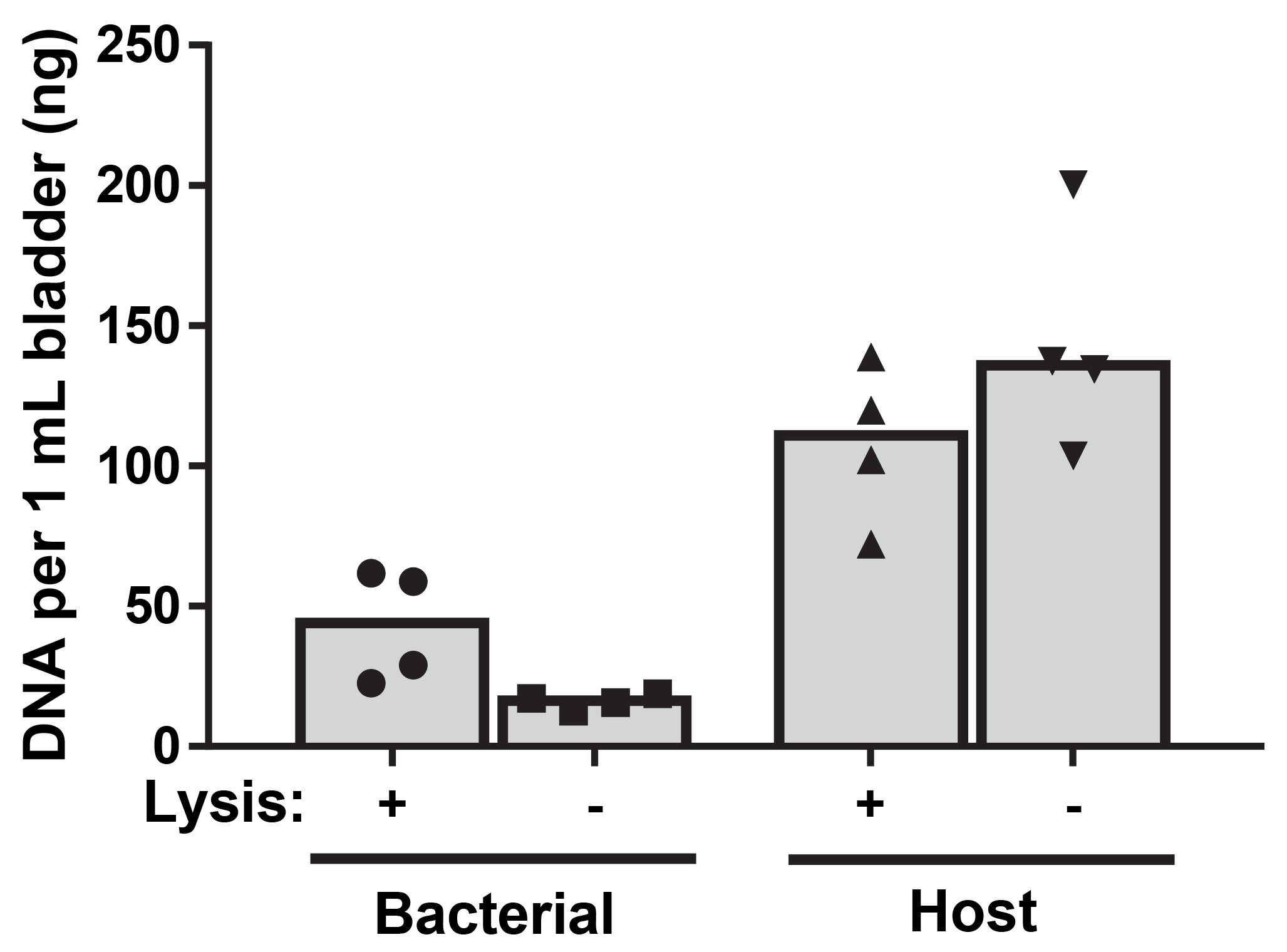

Supplement: FIG S5 [file mbo001183754sf5.tif]

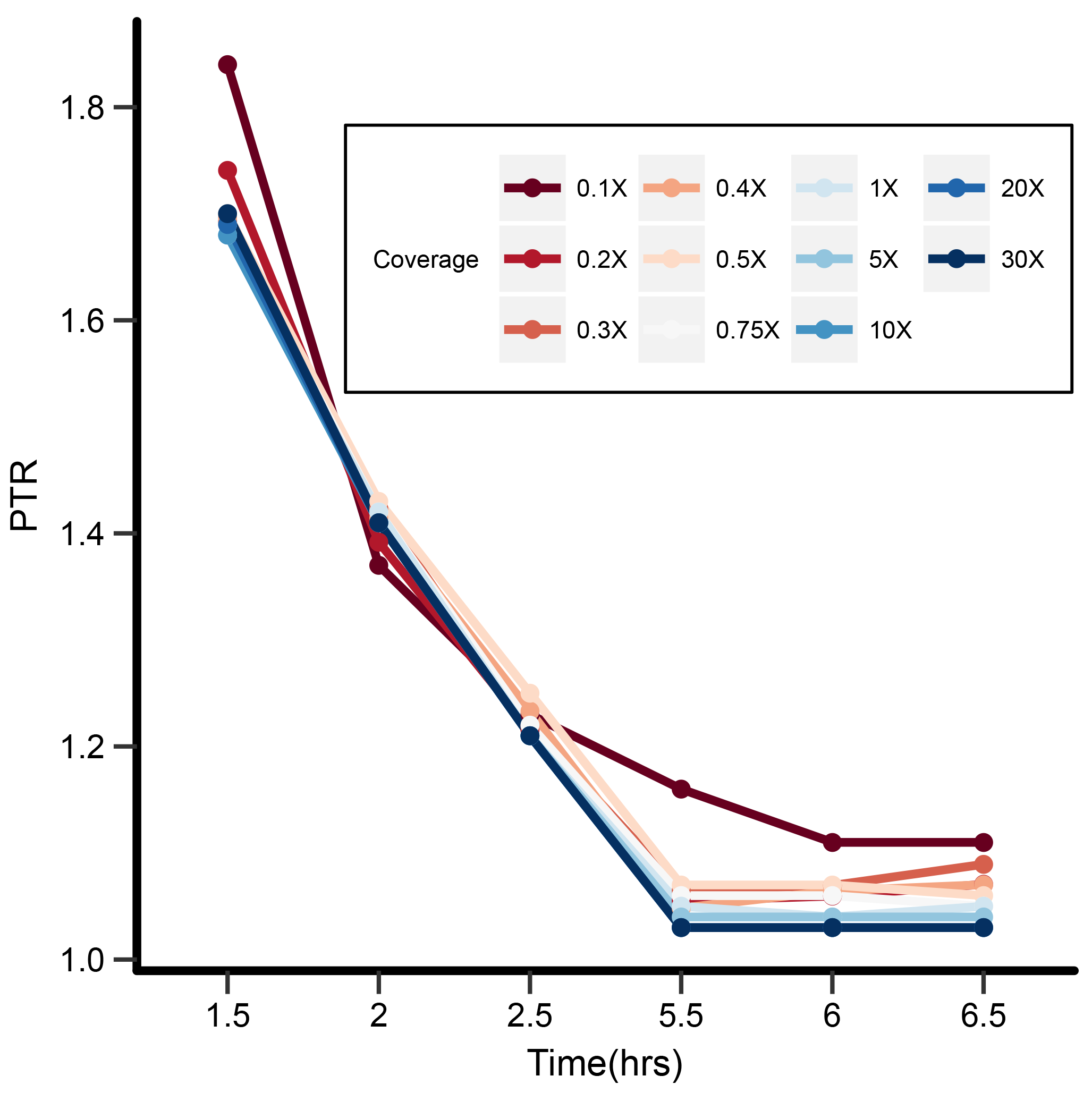

Supplement: FIG S6 [file mbo001183754sf6.tif]

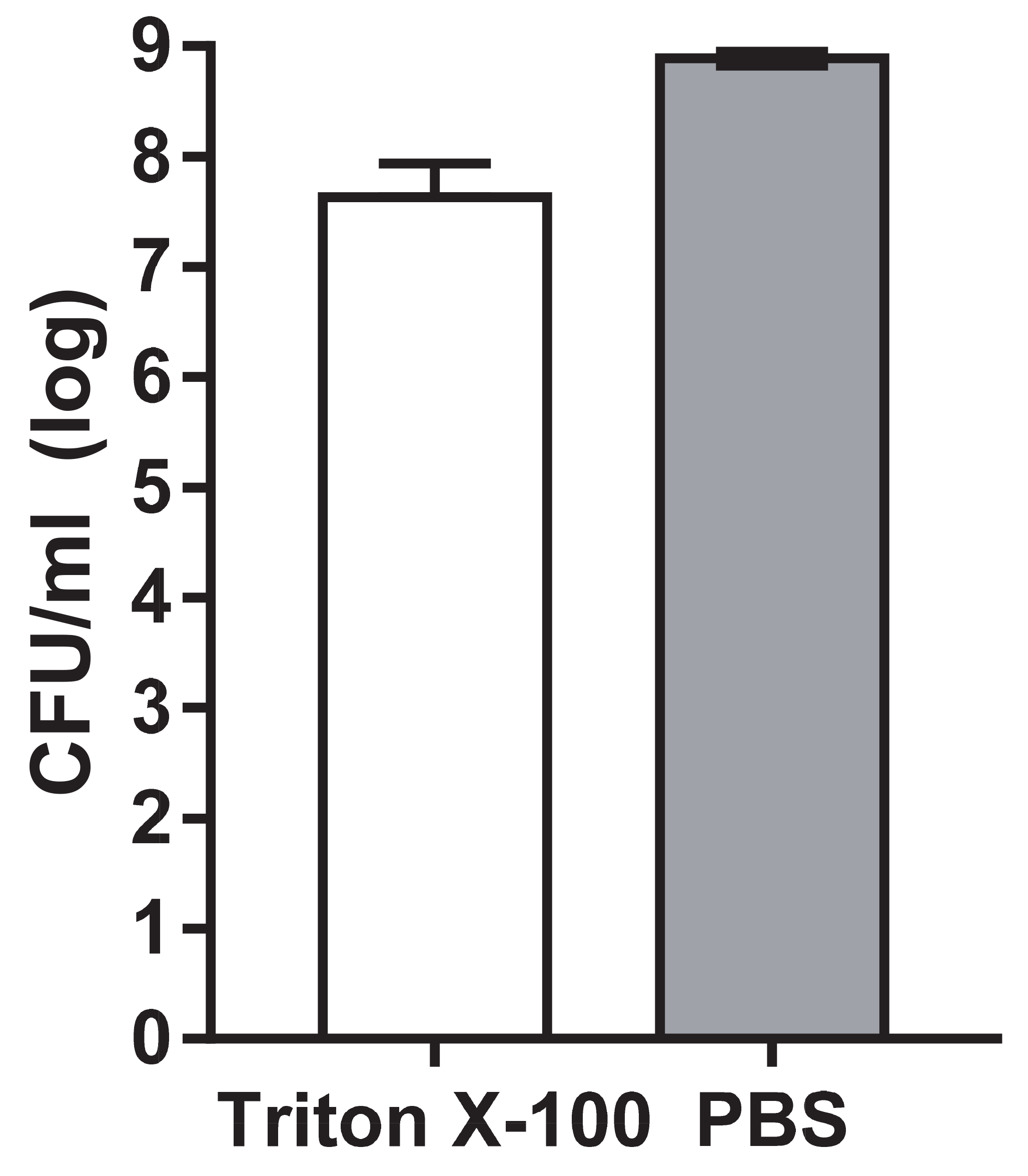

Supplement: FIG S7 [file mbo001183754sf7.tif]

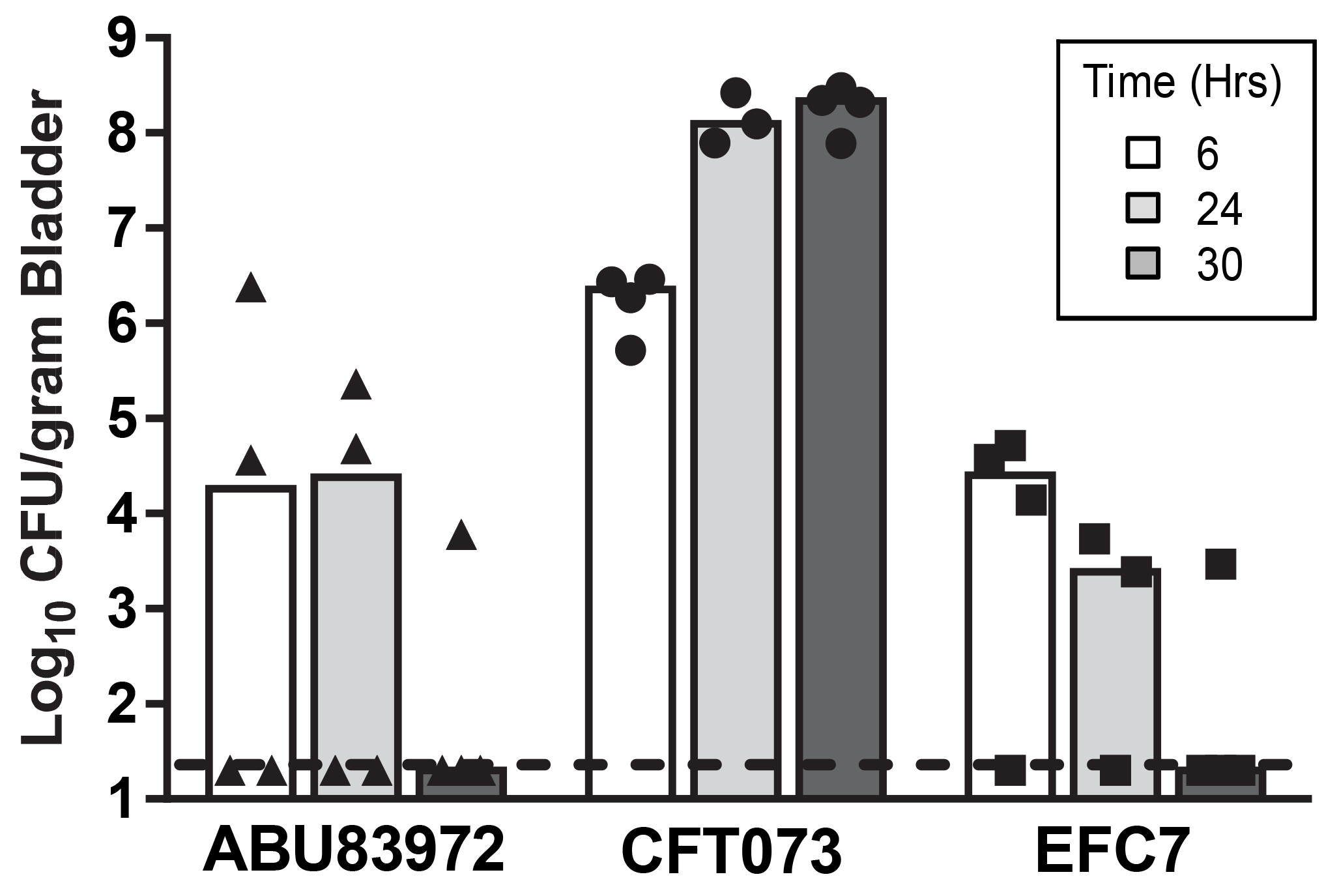

Supplement: FIG S8 [file mbo001183754sf8.tif]

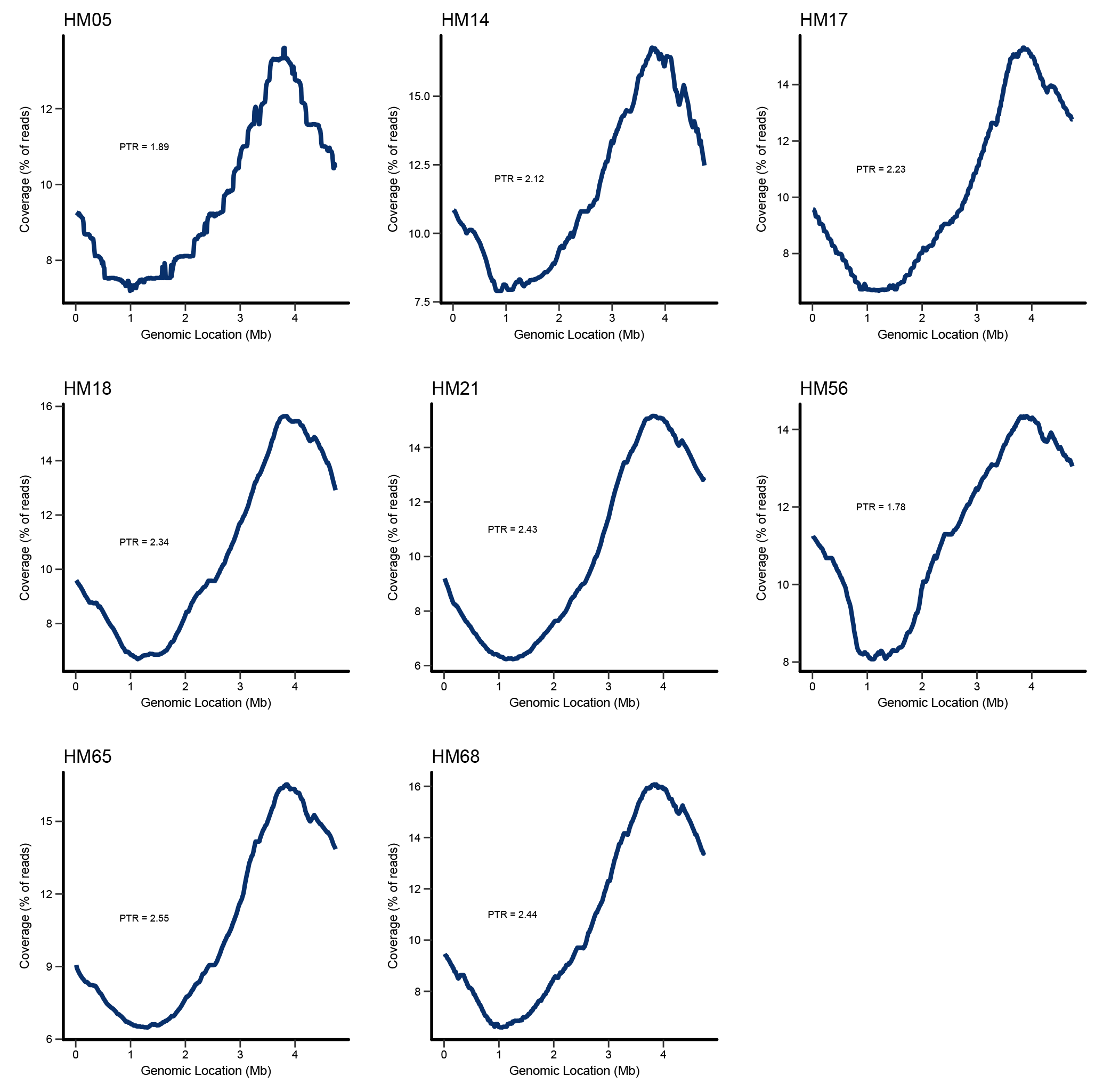

Supplement: FIG S9 [file mbo001183754sf9.tif]
