# Supplementary material for: Rapid Growth of Uropathogenic Escherichia coli during Human Urinary Tract Infection
Source: mBio. 2018 Mar 6;9(2):e00186-18. doi: 10.1128/mBio.00186-18 (PMC5844997; doi:10.1128/mBio.00186-18)
Supplement: TEXT S1 [file mbo001183754s1.docx]

**SUPPLEMENTAL MATERIAL**

**Materials and Methods**

**Ethics statement.** Urine collection was performed as approved by the University of Michigan Institutional Review Board (HUM00004949). Informed consent was obtained from women attending the University Health Services with symptoms of cystitis as approved by the Michigan Institutional Review Board (HUM00029910) (1). All animal protocols were approved by the Institutional Animal Care and Use Committee (IACUC) at the University of Michigan Medical School (PRO00005052), in accordance with the Office of Laboratory Animal Welfare (OLAW) and the United States Department of Agriculture (USDA), as well as guidelines specified by the Association for Assessment and Accreditation of Laboratory Animal Care, International (AAALAC, Intl.). Mice were anesthetized with a weight-appropriate dose (0.1 ml/ 20 gm) of ketamine/xylazine (80-120 mg/kg ketamine and 5-10 mg/kg xylazine) by IP injection. Mice were euthanized by inhalant anesthetic overdose followed by vital organ removal.

**Bacterial strains, plasmids, and culture conditions.** The UPEC strains used in this study were *E. coli* CFT073, UTI89, and 536. Strains CFT073 (2) and 536 (3) are archetypal pyelonephritis isolates recovered from hospitalized patients with acute pyelonephritis, while strain UTI89 (4) was isolated from a patient with an acute bladder infection. *E. coli* commensal fecal strains (EFC1, EFC2, EFC4, and EFC7 (2)) and *E. coli* asymptomatic bacteriuria strains (PUTS37, PUTS58, PUTS59 (5) and ABU83972 (6)) were also used in this study. Genomic DNA of eight UPEC strains was isolated and sequenced from the urine of women attending the University Health Service for the symptoms of cystitis: HM05, HM14, HM17, HM18, HM21, HM56, HM65, and HM68. Where necessary, strains were routinely grown aerobically at 37˚C in lysogeny broth (10 g/L tryptone, 5 g/L yeast extract, 0.5 g/L NaCl) or on LB solidified with 1.5% agar. For plasmid segregation experiments, cultures were supplemented with 1% (wt/vol) L-arabinose to allow plasmid replication and kanamycin (25 μg/ml) for plasmid selection. Plasmid purification and transformation were performed using standard methods (7). For *in vitro* PTR experiments strains were also grown in terrific broth (12 g/L tryptone, 24 g/L yeast extract, 12.8 ml/L 100% glycerol, 17 mM KH_2_PO_4_, 72 mM K_2_HPO_4_ 2 mM MgSO_4_) and M9 minimal medium (2 mM MgSO_4_, 0.1 mM CaCl_2_, 500 mL/L M9 salts (gibco)).

**Segregation of plasmid pGTR902 *in vitro*.** Marker plasmid pGTR902 was constructed for arabinose-inducible replication, a condition not present in the murine host, and was previously used to examine the *in vivo* growth rate of clinical and environmental strains of *Vibrio vulnificus* in a mouse model of subcutaneous skin lesion (8).

*E. coli* strains transformed with pGTR902 were grown overnight with aeration at 37˚C in LB with 1% L-arabinose and kanamycin (25 μg/ml). Bacteria were harvested by centrifugation (5000 x *g*, 10 min), washed three times, resuspended in sterile phosphate-buffered saline (PBS), and diluted 1:1000 in LB with or without 1% L-arabinose. Cultures were grown with aeration at 37˚C, and samples were taken at the start of the experiment and hourly thereafter, and plated on LB agar and LB agar containing 1% (wt/vol) L-arabinose and kanamycin (25 μg/ml) to quantify the total number of bacteria and the number of bacteria that retained pGTR902, respectively. Samples were plated using an Autoplate 4000 spiral plater (Spiral Biotech) and enumerated using a QCount automated plate counter (Spiral Biotech). The number of copies of pGTR902 per cell was estimated by [number of pGTR902-containing bacteria at stationary phase under conditions that are not permissive for plasmid replication (LB without L-arabinose or kanamycin)]/[ number of pGTR902-containing bacteria at T=0].

**Murine model of ascending UTI.** Six- to eight-week-old female C57BL/6 mice (Envigo) were infected as previously described (9, 10). Briefly, bacteria grown with aeration at 37˚C in LB, were harvested by centrifugation, resuspended in sterile PBS, adjusted to OD_600_=4.0 (~2 x 10^9^ CFU/ml), and further diluted where indicated. Mice were transurethrally inoculated with 50 μl of the bacterial suspension (containing 10^8^ CFU) over a 30-sec period. At 1, 2, 4, 6, 24, 30 or 48 hpi, bladder and kidneys were removed, homogenized in PBS using a GLH homogenizer (OMNI International), and plated onto LB agar using an Autoplate 4000 spiral plater (Spiral Biotech) and enumerated using a QCount automated plate counter (Spiral Biotech).

**Segregation of plasmid pGTR902 *in vivo*.** Mice were transurethrally inoculated as described above. Briefly, bacteria transformed with pGTR902 were grown with aeration at 37˚C in LB with 1% L-arabinose and kanamycin (25 μg/ml), harvested by centrifugation, washed three times with sterile PBS, and adjusted to an estimated cell density of 2 x 10^9^ CFU/ml. At 6 hpi, bladder and kidneys were removed, homogenized, and plated onto LB agar with and without 1% L-arabinose and kanamycin (25 μg/ml).

**Estimation of *in vivo* growth rate via plasmid segregation.** Calculations used to determine *in vivo* number of generations have been described previously (8). Briefly, growth proportion: [pGTR902-containing bacteria recovered from the infected murine bladder (CFU on LB agar supplemented with L-arabinose and kanamycin)] – [total number of bacteria recovered (CFU on plain LB agar)]. The number of pGTR902-containing bacteria/g tissue was normalized by plasmid copy number for each specific *E. coli* strain (calculated using the *in vitro* segregation assay). Number of generations: [growth proportion]/[-log_10_2]. In addition, we have made the logical extension of existing formulas to determine the doubling time via the formula [minutes post-inoculation]/[number of generations].

**Estimation of *in vitro* and *in vivo* growth rate via peak to trough ratios (PTR).** The technique has been used recently to compare the relative growth rates of multiple bacterial species simultaneously in microbiome studies (11-14) and is described briefly here. Growth rate can be estimated by calculating PTR for bacteria growing *in vitro* and *in vivo*. For experiments measuring growth rate in LB, terrific broth, or M9 supplemented with 0.4% glucose via PTR, biological replicates were grown with aeration at 37˚C in LB with 1% L-arabinose and kanamycin (25 μg/ml), centrifuged, washed three times with sterile PBS, and back-diluted 1:100 into LB medium. Samples were taken at three time points during exponential phase (30 min intervals for LB and terrific broth, 1 hr intervals for M9 supplemented with 0.4% glucose), pelleted, and genomic DNA was harvested with DNeasy blood and tissue kit (Qiagen). Measurement of growth rate in human urine was performed as above using filter-sterilized mid-stream clean catch human urine pooled from at least three donors and samples were taken at 1 hr intervals 3-8 hrs post inoculation.

To determine the growth rate during murine UTI, bladders were infected, harvested 6 hpi and homogenized as above. Urine from infected mice was collected and pooled 30 min prior to and immediately before sacrifice. Homogenized bladders were subjected to differential lysis of host and bacterial cells to enrich for bacterial genomic DNA as described in (15). Briefly, homogenates were passed through a 40 µm nylon cell strainer and the flow-through was treated with 2 M Na_2_CO_2_ pH 9.8, 1% Triton-X100 before neutralizing in 1 M Tris-HCl, pH4.5. Unlysed bacteria were pelleted at 5,000 x *g* for 5 min and bacterial genomic DNA harvested by the CTAB method. To determine the effect of lysis buffer on bacterial viability, saturated cultures of CFT073, EFC7, and ABU83972 grown in LB medium were treated with either PBS or with 2 M Na_2_CO_2_ pH 9.8, 1% Triton-X100 before neutralizing in 1 M Tris-HCl, pH4.5. Bacteria were then plated for viable counts. Treatment with lysis buffer resulted in a 1.3 log_10_ decrease in CFU (Fig. S7). Estimation of growth rate during human uncomplicated UTI was performed using mid-stream clean catch urine, stabilized with *RNA-Protect* (Qiagen)*,* harvested from patients with symptoms of cystitis (1). Urine samples were subjected to centrifugation, and genomic DNA was extracted from the resulting pellets using the CTAB method.

Genomic DNA from all sample types were fragmented, barcoded, multiplexed up to 22 samples per flow cell lane and subjected to V4 single end 50 HiSeq-2500 High-Output Illumina sequencing at the University of Michigan DNA core facility. The quality of raw reads was assessed with FastQC (16). Trimmomatic (17) was used for trimming the adapter sequences and low quality bases, using a sliding window of 4 bp with average quality threshold parameter set to 20, and other parameters set to default. The filtered reads were then mapped to its respective reference genome using Bowtie2 (18) aligner with parameters set to align entire read end-to-end and report only one unique alignment per read (-k 1 --end-to-end). The alignments were sorted and converted to BAM format with SAM tools and PCR duplicates were removed using Picard. The bedtools genomecov utility was used to report coverage depth at each position from BAM alignments. A smoothing filter as described in (14) was applied to the raw coverage by considering a moving sum with window size of 10 Kbp and a slide of 100 bp, followed by a moving median with window size of 10K bins and a slide of 100 bins. An outlier filter was applied on the resulting bins where empty bins were removed from final PTR calculation. PTRs were calculated using the peak and trough location, that is, maximum and minimum values from the resulting smoothed coverage. The growth rates were inferred from the linear model (Fig. 3D) and doubling time were calculated using the following equation: doubling time = ln(2) / growth rate.

**Expression of type 1 fimbriae.** To investigate production of type 1 fimbriae in UPEC, fecal and asymptomatic strains, the ability of these strains to agglutinate yeast (*Sacchoromyces cerevisiae*) was qualitatively observed. CFT073, EFC7, and ABU83972 were cultured in LB medium at 37˚C without aeration 48 hrs. Bacterial suspensions were concentrated to OD_600_=6.0, mixed in equal volume with a suspension of 3% yeast in PBS and incubated on ice for 30 min. Titers were determined as the last bacterial dilution to maintain agglutination of the yeast.

**REFERENCES**

1. Subashchandrabose S, Hazen TH, Brumbaugh AR, Himpsl SD, Smith SN, Ernst RD, Rasko DA, Mobley HL. 2014. Host-specific induction of Escherichia coli fitness genes during human urinary tract infection. Proc Natl Acad Sci U S A 111:18327-32.

2. Mobley HL, Green DM, Trifillis AL, Johnson DE, Chippendale GR, Lockatell CV, Jones BD, Warren JW. 1990. Pyelonephritogenic *Escherichia coli* and killing of cultured human renal proximal tubular epithelial cells: role of hemolysin in some strains. Infect Immun 58:1281-9.

3. Berger H, Hacker J, Juarez A, Hughes C, Goebel W. 1982. Cloning of the chromosomal determinants encoding hemolysin production and mannose-resistant hemagglutination in *Escherichia coli*. J Bacteriol 152:1241-7.

4. Mulvey MA, Schilling JD, Hultgren SJ. 2001. Establishment of a persistent *Escherichia coli* reservoir during the acute phase of a bladder infection. Infect Immun 69:4572-9.

5. Hooton TM, Scholes D, Stapleton AE, Roberts PL, Winter C, Gupta K, Samadpour M, Stamm WE. 2000. A prospective study of asymptomatic bacteriuria in sexually active young women. N Engl J Med 343:992-7.

6. Andersson P, Engberg I, Lidin-Janson G, Lincoln K, Hull R, Hull S, Svanborg C. 1991. Persistence of Escherichia coli bacteriuria is not determined by bacterial adherence. Infect Immun 59:2915-21.

7. Sambrook J, Fritsch EF, Maniatis T. 1989. Transformation of E. coli by High-voltage Electroporation, 1.75-76. *In* Sambrook J, Fritsch EF, Maniatis T (ed), Molecular cloning: a laboratory manual, 2^nd^ ed. Cold Spring Harbor Laboratory Press.

8. Starks AM, Bourdage KL, Thiaville PC, Gulig PA. 2006. Use of a marker plasmid to examine differential rates of growth and death between clinical and environmental strains of *Vibrio vulnificus* in experimentally infected mice. Mol Microbiol 61:310-23.

9. Hagberg L, Engberg I, Freter R, Lam J, Olling S, Svanborg Eden C. 1983. Ascending, unobstructed urinary tract infection in mice caused by pyelonephritogenic *Escherichia coli* of human origin. Infect Immun 40:273-83.

10. Johnson DE, Lockatell CV, Russell RG, Hebel JR, Island MD, Stapleton A, Stamm WE, Warren JW. 1998. Comparison of *Escherichia coli* strains recovered from human cystitis and pyelonephritis infections in transurethrally challenged mice. Infect Immun 66:3059-65.

11. Olm MR, Brown CT, Brooks B, Firek B, Baker R, Burstein D, Soenjoyo K, Thomas BC, Morowitz M, Banfield JF. 2017. Identical bacterial populations colonize premature infant gut, skin, and oral microbiomes and exhibit different in situ growth rates. Genome Research 27:601-612.

12. Olm MR, Butterfield CN, Copeland A, Boles TC, Thomas BC, Banfield JF. 2017. The Source and Evolutionary History of a Microbial Contaminant Identified Through Soil Metagenomic Analysis. mBio 8(1):e01969-16.

13. Brown CT, Olm MR, Thomas BC, Banfield JF. 2016. Measurement of bacterial replication rates in microbial communities. Nat Biotech 34:1256-1263.

14. Korem T, Zeevi D, Suez J, Weinberger A, Avnit-Sagi T, Pompan-Lotan M, Matot E, Jona G, Harmelin A, Cohen N, Sirota-Madi A, Thaiss CA, Pevsner-Fischer M, Sorek R, Xavier RJ, Elinav E, Segal E. 2015. Growth dynamics of gut microbiota in health and disease inferred from single metagenomic samples. Science 349:1101-1106.

15. Trung NT, Hien TTT, Huyen TTT, Quyen DT, Van Son T, Hoan PQ, Phuong NTK, Lien TT, Binh MT, Van Tong H, Meyer CG, Velavan TP, Song LH. 2016. Enrichment of bacterial DNA for the diagnosis of blood stream infections. BMC Infectious Diseases 16:235.

16. Andrews S. 2010. FastQC: a quality control tool for high throughput sequence data.

17. Bolger AM, Lohse M, Usadel B. 2014. Trimmomatic: a flexible trimmer for Illumina sequence data. Bioinformatics 30:2114-2120.

18. Langmead B, Salzberg SL. 2012. Fast gapped-read alignment with Bowtie 2. Nat Meth 9:357-359.
